# Monopolar radiofrequency for dermal temperature regulation and remodeling: A porcine model study

**DOI:** 10.1111/jocd.16495

**Published:** 2024-07-24

**Authors:** Chidae Park, Jumi Hong, Hye Guk Ryu, Seokhong Kim, Jinyoung Park, Kyung Kim, Jongah Won, Jungmi Lee, Soo Il Chun

**Affiliations:** ^1^ RA Team Lutronic Corporation Goyang Korea; ^2^ Clinical Development Team Lutronic Corporation Goyang Korea; ^3^ R&D Department Lutronic Corporation Goyang Korea; ^4^ Chunsooil Skin Clinic Seoul Korea

**Keywords:** collagen, dermal remodeling, dermal temperature, neocollagenesis, noninvasive monopolar radiofrequency

## Abstract

**Background:**

Noninvasive monopolar radiofrequency (NMRF) is widely used for dermal and subdermal volumetric heating, yet detailed research on its effects on dermal temperature is scarce.

**Aims:**

This study evaluates the impact of NMRF on dermal temperature and its potential for dermal remodeling using a porcine model.

**Methods:**

Noninvasive monopolar radiofrequency was applied to porcine skin with temperature monitoring via optic fiber technology and forward‐looking infrared thermal imaging. Safety was evaluated using nitro blue tetrazolium chloride assessments, and effectiveness was determined through histological examinations before and after treatment.

**Results:**

Noninvasive monopolar radiofrequency treatment in a porcine model achieved effective dermal remodeling with no thermal damage, recording peak temperatures of 50°C, 60°C, and 70°C. Histological analysis showed increased collagen density, indicating successful tissue remodeling.

**Conclusion:**

Noninvasive monopolar radiofrequency is effective in delivering controlled dermal heating and enhancing collagen synthesis, promoting safe and efficient skin tightening and dermal remodeling in a porcine model. It presents a viable option for skin rejuvenation therapies.

## 1 INTRODUCTION

Over the past years, notable advancements in the development of laser and energy‐based devices have greatly influenced aesthetic dermatology. Radiofrequency (RF) technology, particularly monopolar RF, has become crucial for skin rejuvenation due to its efficacy, minimal side effects, and short downtime. Monopolar RF therapy delivers heat evenly to the dermal layers, promoting immediate collagen structure changes, leading to skin tightening and firming. Over time, the new collagen bundles are remodeled and reoriented, enhancing this firming effect with tight arrangement of collagen that can continue for months after treatment.^1^, ^2^

The first monopolar RF device received FDA approval in 2002 for treating facial wrinkles.^3^, ^4^ Subsequent studies confirmed its safety and efficacy, resulting in FDA approval for additional uses, including nonsurgical skin tightening, treatment of lower facial laxity, scar treatment, and body contouring.^5^, ^6^, ^7^

Unlike selective photothermolysis, which targets specific skin chromophores, RF devices generate heat through tissue impedance, converting electrical current to thermal energy based on the tissue's properties. Optimal thermal effects in the dermal region are crucial for effective monopolar RF therapy. Research indicates collagen remodeling occurs between 42°C and 65°C, and lipolysis and sculpting between 65°C and 70°C, with nerve damage risk at temperatures around 85°C.^8^ Despite extensive research, detailed measurements of dermal tissue temperatures and their effects remain limited, especially for monopolar RF. Our study investigates the temperature dynamics induced by monopolar RF in the dermal region, assessing changes in collagen and overall tissue response. We also evaluate safety parameters associated with the thermal effects of monopolar RF to ensure its effective and safe clinical application. This research aims to provide deeper insights into the mechanisms by which monopolar RF rejuvenates the skin.

## 2 MATERIALS AND METHODS

### 2.1 Devices and experimental design

An XERF system (Cynosure Lutronic Inc.), a device based on noninvasive monopolar RF technology engineered to deliver heat to the dermis and beyond while employing a cooling system to protect the epidermis, was used in this study. Standard female pigs (*Sus scrofa*), aged 2–3 months and weighing between 24 and 28 kg, were used as the experimental subjects. Noninvasive monopolar radiofrequency (NMRF) treatments were applied to the dorsal surface of the animals. The study adhered to institutional guidelines for standard operating procedures, providing the animals with environmental enrichment and veterinary oversight. The Institutional Animal Care and Use Committee (IACUC) of CRONEX Corporation approved all experimental protocols under authorization number CRONEX‐IACUC: 202305004.

### 2.2 Histopathological analysis

Paired 8‐mm punch biopsy specimens were collected from the test area. Histology evaluations were conducted at baseline and 30 days post‐treatment with Masson's trichrome (MT) stain or nitro blue tetrazolium chloride (NBTC) stain. Samples were fixed in 4% paraformaldehyde for 24 h, paraffin‐embedded, and sectioned at 5 μm. Sections were stained with MT to identify collagen fibers and with NBTC to assess cellular viability and analyzed using an Olympus DP72 camera and image analysis software.

### 2.3 Temperature evaluation in the dermis

**FIGURE 1:**
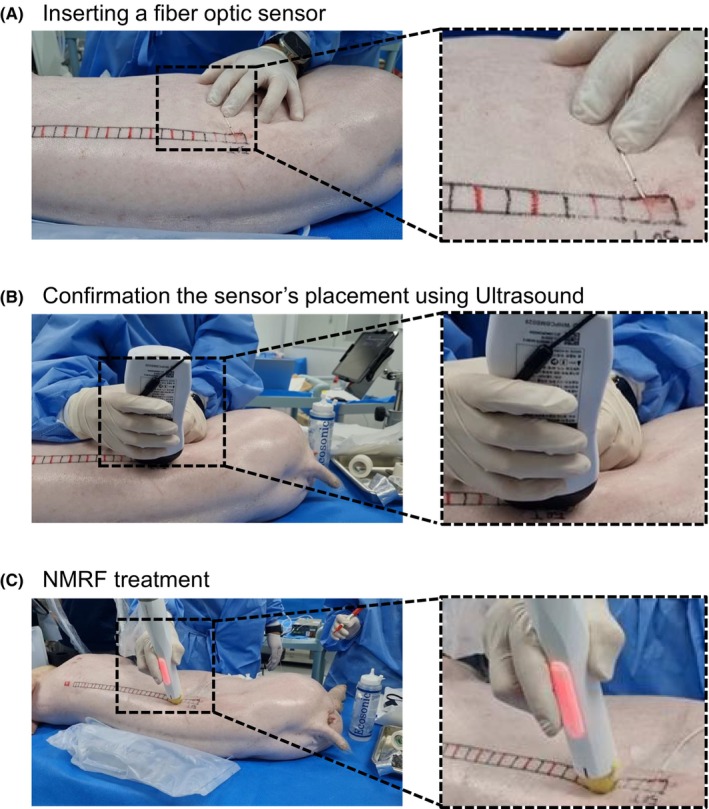
Performance evaluation of dermal temperature and ultrasound measurement for noninvasive monopolar radiofrequency (NMRF) treatment. These images depict the methodology used to assess the safety and effectiveness of the NMRF device, through preclinical testing on a pig model. The process included several key steps: (A) inserting a fiber optic sensor to measure temperature in the dermal region, (B) using ultrasound to confirm the sensor's correct placement at the appropriate dermal depth, and (C) conducting the NMRF treatment. This sequence was critical for collecting precise data on the device's ability to maintain controlled temperatures.

We evaluated the temperature changes within the dermal layer upon exposure to NMRF energy with a Fiber Optic Thermometer (FOB100; OMEGA Engineering Inc., USA) (Figure S1A). To accurately measure the temperature within the dermis, a fiber optic temperature sensor probe was inserted into the target skin area prior to RF application (Figure 1A). Subsequently, the placement of the temperature sensor within the dermal layer was verified using an ultrasonic diagnostic device (SonoMe, Bionet, Jane 21–4698), ensuring it was positioned correctly by examining the depth of insertion (Figure 1B). Following the establishment of output parameters, NMRF energy was applied, and dermal temperature measurements were conducted with the fiber optic thermometer (Figure 1C).

### 2.4 Surface temperature measurement

The objective of this experiment was to confirm that the device maintains a safe operational temperature, ensuring that it does not exceed the threshold that might cause thermal damage to the skin surface. To accurately monitor the surface temperature throughout the procedure, we used an infrared thermal imaging camera (Forward Looking infrared [FLIR] A325, FLIR System Inc., Sweden). This camera is known for its precision in detecting and recording subtle variations in surface temperature. Measurements were taken continuously during the treatment to ensure that the temperature did not exceed 43°C, thereby verifying the device's safety under operational conditions.

## 3 RESULTS

### 3.1 Real‐time thermal changes in porcine dermis

**FIGURE 2:**
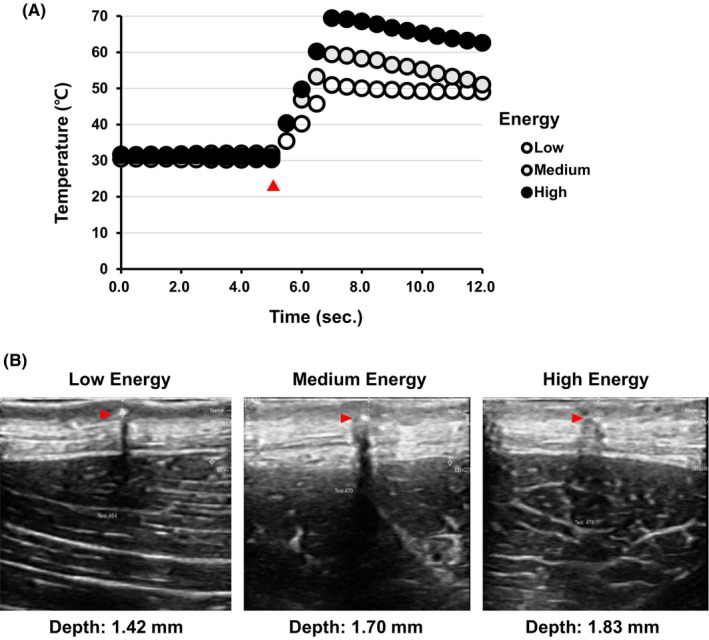
Deep tissue temperature measurements and ultrasound‐guided fiber placement. (A) Real‐time dermal temperature monitoring: This panel displays a graphical representation of dermal temperature variations under different energy levels. The temperature of the dermal region was confirmed to reach 50°C after low‐energy treatment, 60°C after medium‐energy treatment, and 70°C following high‐energy treatment. Red arrowheads indicate the time points at the start of NMRF treatment. (B) It includes ultrasound images to verify the precise placement of thermal fibers in the dermis. Red arrowheads indicate the locations where the thermal fibers are inserted.

Using the optical fiber thermometer, we monitored dermal temperature in real time. We observed that the dermal temperature quickly reached a peak of 50°C at low energy settings, 60°C at medium energy settings, and then gradually declined. At high energy settings, the temperature rose to about 70°C before slowly decreasing (Figure 2A). Ultrasound imaging verified the placement of the fiber optic sensor within the dermis (Figure 2B). Based on these results, we confirmed that temperatures capable of inducing tissue changes were effectively transmitted to the dermal region.

### 3.2 In vivo skin surface temperature changes and evaluation of histological changes

**FIGURE 3:**
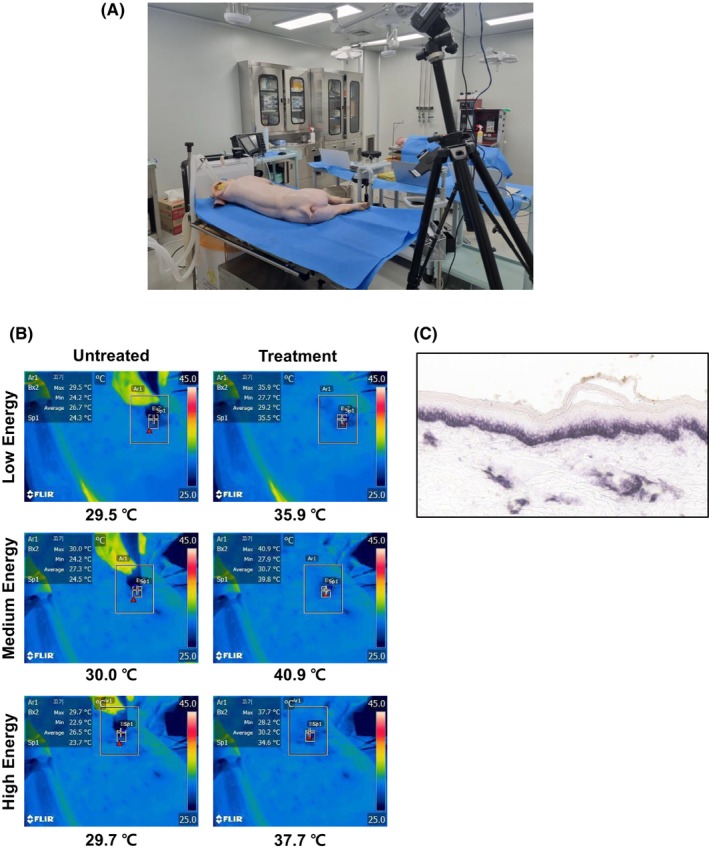
Skin surface temperature monitoring and nitro blue tetrazolium chloride (NBTC) staining for tissue health posttreatment. (A) This image illustrates the methodology for assessing skin surface temperature using a thermal imaging camera, which captures detailed thermal images and temperature data. (B) Thermal imaging photos depicting surface skin temperature before and immediately after posttreatment with the noninvasive monopolar radiofrequency device. Temperatures remained below 43°C across all tested parameters. (C) NBTC staining results indicating healthy tissue status 1‐month posttreatment. The panels demonstrate significant maintenance of healthy tissue architecture, which supports the conclusion that the treated areas have retained their structural integrity.

We confirmed that the heat energy generated was transferred from the skin surface to the dermal region, selectively maintaining a temperature range of 50–60°C, which is the ideal range for collagen restructuring, fibroblast activation, and subsequent remodeling. When delivering heat to the dermis, it is essential to keep skin surface temperatures below 42–45°C to avoid thermal damage to the epidermis.^9^ A previous study noted that when heating of the dermis occurs from 65°C to 75°C at a depth from 3 to 6 mm, it is necessary to cool the epidermis to maintain temperatures between 35°C and 45°C.^10^ Forward Looking infrared imaging was used experimentally to confirm that the surface temperatures remained stable while the dermal temperatures were within 50–70°C (Figure 3A). Measurements indicated that skin surface temperatures did not exceed 42–45°C under test conditions for each group (Figure 3B). In 30 days post‐NMRF treatment, a thermal damage assessment was conducted. Nitro blue tetrazolium chloride‐stained histological sections of porcine skin showed no thermal damage to the dermis or epidermis (Figure 3C), confirming that NMRF generated temperatures that did not cause thermal damage to the skin surface.

### 3.3 Histological change measurement for efficacy evaluation

**FIGURE 4:**
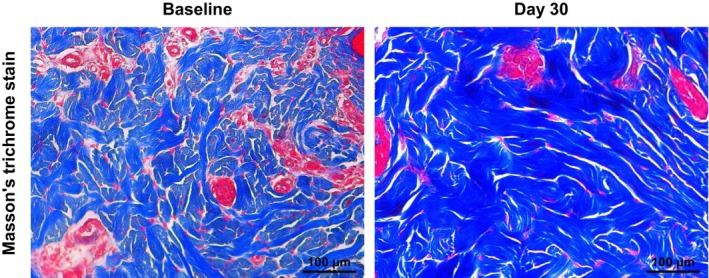
Collagen remodeling assessment following noninvasive monopolar radiofrequency (NMRF) treatment. Masson's trichrome stain used to evaluate the efficacy of the NMRF treatment in inducing significant changes in tissue structure, specifically collagen remodeling in the dermis 30 days posttreatment. Our observations confirmed that beyond merely achieving the desired temperature, the treatment facilitated a denser and thicker arrangement of collagen bundles. This transformation is crucial as it highlights the ultimate goal of the NMRF treatment: To induce beneficial changes in tissue structure, confirming its impact on the dermal matrix.

Numerous studies have shown that NMRF can tighten skin through controlled contraction of dermal collagen and neocollagenesis, without disrupting the epidermis.^11^, ^12^, ^13^ To evaluate whether NMRF's precise temperature delivery could induce tissue changes, including neocollagenesis, we conducted a skin histopathological examination using MT staining. Thirty days posttreatment, an increase in collagen thickness and density within the tissue, was observed, confirming that NMRF effectively delivered the necessary temperatures neocollagenesis, followed by tissue remodeling (Figure 4).

## 4 DISCUSSION

Our study highlights significant advancements in using NMRF for dermal remodeling with a porcine model. In addition to NMRF, other techniques such as fractional laser therapy, which targets microzones in the skin; microneedling, which creates microinjuries to enhance collagen production; and high‐intensity focused ultrasound, which heats deeper skin layers, also promote skin remodeling and neocollagenesis. While each method has its distinct benefits, NMRF is particularly notable for its ability to uniformly distribute heat, ensuring efficient skin rejuvenation with minimal downtime and adverse effects. Moreover, NMRF has been validated for safety and efficiency through various studies, leading to multiple FDA approvals.^14^, ^15^, ^16^ However, detailed analysis of internal tissue temperatures and their effects on the dermis, particularly with monopolar RF, has been lacking.

In this study, the use of a fiber optic thermometer accurately placed through ultrasound imaging enabled monitoring of the effects induced by NMRF, providing accurate measurements of the actual temperature ranges critical for effective tissue remodeling without causing thermal damage. Maintaining subdermal temperatures between 50°C, 60°C, and 70°C during treatment, along with verifying tissue integrity, demonstrated the safety and efficacy of our NMRF system. Histological analysis showed increased collagen density and thickness, indicating successful neocollagenesis, followed by tissue remodeling.

Our investigation addresses the gap in existing research by providing detailed measurements of internal tissue temperatures and their effects on the dermis. This study validates the established therapeutic windows for effective RF treatments and enhances our understanding of thermal dynamics in tissue. While these are significant strengths, there is still a need for a comprehensive evaluation of temperatures across various skin layers in future studies. Particularly, as many studies mention the potential for NMRF to transfer heat to the subcutaneous layer,^1^, ^17^ it is crucial to integrate temperature measurements across the epidermis, dermis, and subcutaneous regions for a more complete understanding.

In conclusion, our study demonstrates that our NMRF system can deliver precise temperatures to induce tissue changes in the dermal region while protecting the skin surface. Our findings show this method effectively stimulates neocollagenesis, offering a comprehensive approach to skin rejuvenation and structural remodeling in aesthetic dermatology.

## AUTHOR CONTRIBUTIONS

Chidae Park, Jumi Hong, and Soo il Chun designed the study. Chidae Park, Jumi Hong, Hye Guk Ryu, Seokhong Kim, Jinyoung Kim, and Kyung Kim performed the experiments. Chidae Park, Jumi Hong, Hye Guk Ryu, Jongah Won, Jungmi Lee, and Soo il Chun depict the picture in the article. Chidae Park, Jumi Hong, Hye Guk Ryu, Jongah Won, Jungmi Lee, and Seokhong Kim analyzed the data and wrote the manuscript. All authors approved the manuscript.

## FUNDING INFORMATION

The author received no financial support for the research, authorship, and publication of this article.

## CONFLICT OF INTEREST STATEMENT

The authors have declared that no competing interest exists.

## ETHICS STATEMENT

All experimental procedures were in accordance with the guidelines of Laboratory Animal Manual of National Institute of Health Guide for the Care and Use of Animals. They were approved by the Institutional Animal Care and Use Committee of CRONEX Corporation (No. CRONEX‐IACUC: 202305004).

## Supporting information


Figure S1.

## Data Availability

The data that support the findings of this study are available from the corresponding author upon reasonable request.
