# Supplementary figures and images for: Monopolar radiofrequency for dermal temperature regulation and remodeling: A porcine model study
Source: J Cosmet Dermatol. 2024 Jul 24;23(12):3955–60. doi: 10.1111/jocd.16495 (PMC11626309; doi:10.1111/jocd.16495)

# Supplementary Fig. 1

A

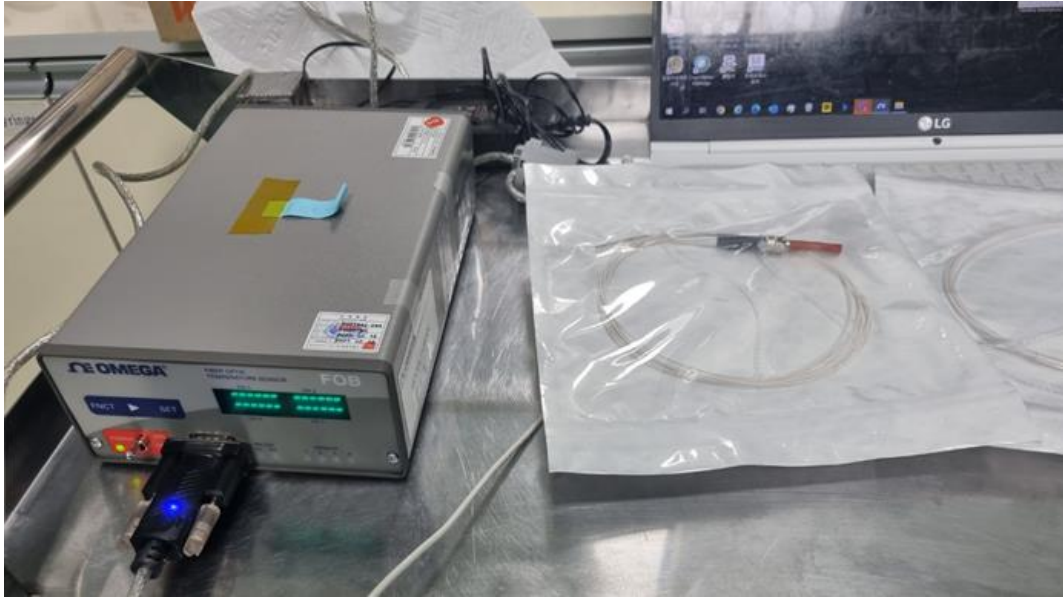

Supplement: Supplementary file 1 — Figure S1. [file JOCD-23--s001.pdf]
